# How reliable are ADC measurements? A phantom and clinical study of cervical lymph nodes

**DOI:** 10.1007/s00330-017-5265-2

**Published:** 2018-02-23

**Authors:** Bastien Moreau, Antoine Iannessi, Christopher Hoog, Hubert Beaumont

**Affiliations:** 1 Department of Radiology, Centre Antoine Lacassagne, 06100 Nice, France; 2 Research and Development Department, Median Technologies, Les deux arcs - 1800 route des crêtes - Bat. B, 06560 Valbonne, France

**Keywords:** Magnetic resonance imaging, Diffusion, Biomarkers, Lymph, Quantitative evaluation

## Abstract

**Objective:**

To assess the reliability of ADC measurements in vitro and in cervical lymph nodes of healthy volunteers.

**Methods:**

We used a GE 1.5 T MRI scanner and a first ice-water phantom according to recommendations released by the Quantitative Imaging Biomarker Alliance (QIBA) for assessing ADC against reference values. We analysed the target size effect by using a second phantom made of six inserted spheres with diameters ranging from 10 to 37 mm. Thirteen healthy volunteers were also scanned to assess the inter- and intra-observer reproducibility of volumetric ADC measurements of cervical lymph nodes.

**Results:**

On the ice-water phantom, the error in ADC measurements was less than 4.3 %. The spatial bias due to the non-linearity of gradient fields was found to be 24 % at 8 cm from the isocentre. ADC measure reliability decreased when addressing small targets due to partial volume effects (up to 12.8 %). The mean ADC value of cervical lymph nodes was 0.87.10^-3^ ± 0.12.10^-3^ mm^2^/s with a good intra-observer reliability. Inter-observer reproducibility featured a bias of -5.5 % due to segmentation issues.

**Conclusion:**

ADC is a potentially important imaging biomarker in oncology; however, variability issues preclude its broader adoption. Reliable use of ADC requires technical advances and systematic quality control.

**Key Points:**

• *ADC is a promising quantitative imaging biomarker.*

• *ADC has a fair inter-reader variability and good intra-reader variability.*

• *Partial volume effect, post-processing software and non-linearity of scanners are limiting factors.*

• *No threshold values for detecting cervical lymph node malignancy can be drawn.*

**Electronic supplementary material:**

The online version of this article (10.1007/s00330-017-5265-2) contains supplementary material, which is available to authorized users.

## Introduction

Recent advances in medical imaging technology and drug therapeutics have accelerated the emergence of new quantitative imaging biomarkers (QIB) [1, 2]. The multiplication of these QIBs is unfortunately not always accompanied by stringent validations establishing that QIBs are well designed to characterize a disease and its changes with therapy. This lack of validation creates a situation where QIBs are routinely used but with limited knowledge of their performances, precluding a larger adoption in clinical trials.

Apparent diffusion coefficient (ADC) can quantify the level of free water diffusion restricted by an increase in tissue cellularity. Applications of ADC in cancer imaging has motivated intensive research and ADC is now one of the main QIBs derived from diffusion MRI.

Several studies have documented the incremental value of ADC assessment as a complement or substitute to standard sequences for the detection of malignant tumours [3], the degree of malignancy [4, 5] or to evaluate response to treatment [6–8].

Since lymph node involvement is pivotal in oncological imaging [9], ADC has been tested for its detection of malignant adenomegalies [10, 11]. Results are discordant [12, 13].

Previous literature comprises heterogeneous studies protocols and results [14]. Several sequential unitary processes are necessary to output an ADC assessment, the lack of reliability of any of these unitary processes is likely to degrade the final ADC assessment. It is therefore particularly relevant to study if ADC qualifies as a quantitative biomarker.

Over the last decade, a multidisciplinary community has organized retrospective investigations of QIBs starting by documenting methodologies [2]. In 2007, the Radiological Society of North America (RSNA) launched QIBA (Quantitative Imaging Biomarker Alliance [15]), a specialized working group aiming at improving the value and usefulness of QIBs in reducing variability across devices, patients, and practices.

One of QIBA aims consists in releasing ‘Profiles’, which are documents standardizing imaging protocols to obtain optimal, reliable and reproducible biomarker measures according to the current state of the art. The QIBA diffusion imaging profile is still a work in progress [16].

QIBA also proposes a standardized protocol for quality control in diffusion imaging, using a diffusion phantom [17, 18] consisting of a volume of 0 °C stabilized water as the reference value for ADC assessment [19, 20].

The main objective of this study was to evaluate the variability of ADC measurements in vitro on a phantom and in vivo on cervical lymph nodes. The secondary objective was to understand and quantify ADC measurement errors, in view of correcting them in future studies.

## Methods

We first tested QIBA metrics for quality control (QC) of ADC image quality, and then performed a reliability analysis of ADC measurements. Finally we measured ADC values of cervical lymph nodes in healthy volunteers.

This prospective study was conducted at the Centre Antoine Lacassagne, cancer centre in Nice, France, between March and November 2016. We used a GE MRI scanner 1-5T MR450W and ADW Volume Share 5 4.6 software to process images (GE Healthcare).

### Quality control test

**Fig. 1:**
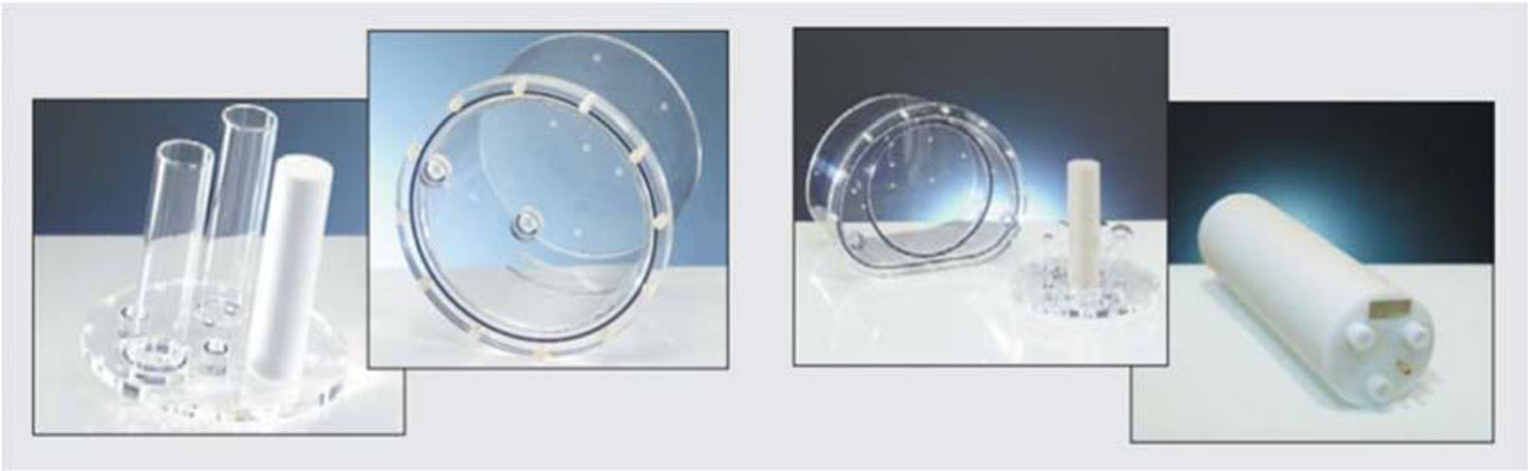
Phantoms used in the study. **Left** ICEWATER phantom filled with 0° C water (*DIN 6858-1, PTW, Freiburg, Germany)*. **Right** SPHERE phantom at room temperature featuring spheres of various sizes between 10- and 37-mm diameters (NEMA NU2-2012, PTW, Freiburg, Germany)

We used a DIN 6858-1 PET-CT phantom (PTW) consisting of a cylindrical Plexiglas body filled with a mixture of ice and water. Three smaller cylinders were inserted into the body, one of which was filled with water at 0 °C (Fig. 1, left side).

Homogeneity of temperature inside the cylinder was thermometer-controlled according to the process defined into the QIBA profile to achieve thermal equilibrium (>1 h) over the entire MRI exam period. For each b value, four successive acquisitions spaced in time from more than 12 min were performed, allowing retrospective checks.

The diffusion protocol was 3three directions, DW SS-EPI with b=0, 100, 600, 800 s/mm^2^, TR=9,451 ms, TE=80 ms, Number of average = 2, FOV 320*320 mm, contiguous slice thickness of 4 mm, encoding frequency axis R/L.

**Fig. 2:**
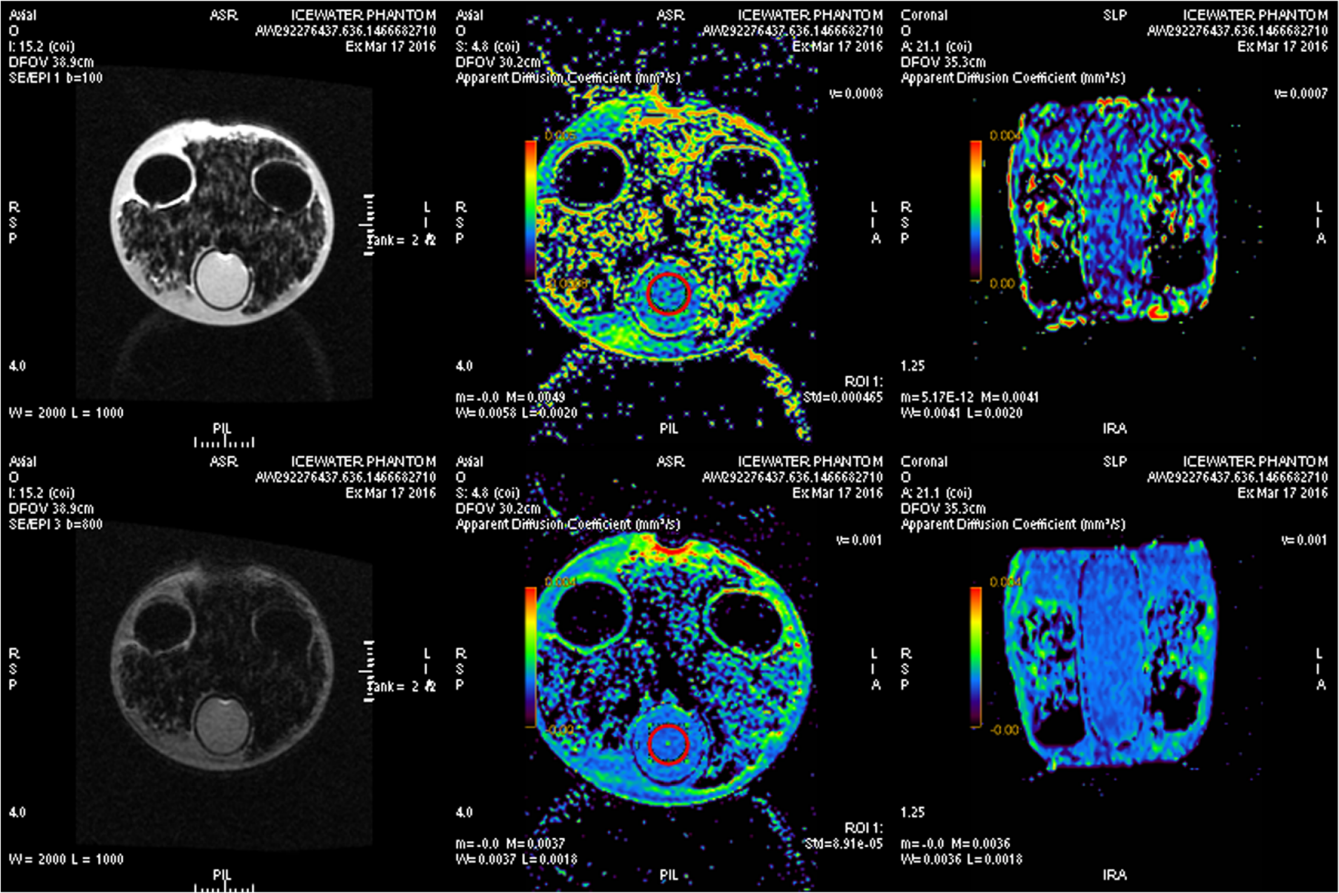
Measurements on the ICEWATER phantom. Imaging of ICEWATER phantom at b0-b100 (**top**) and b0-b800 (bottom). From left to right: Diffusion mapping, axial view of ADC mapping and coronal view of ADC mapping. Red circular regions of interest are set at the centre of the ice water cylinder

Four successive acquisitions were made for each b value, the phantom symmetry axis was laser-centred to the magnetic field positioning the 0 °C water cylinder at the center of the scanner. Acquisitions of the phantom were performed horizontally (x-axis) and vertically (y-axis). We measured circular regions of interest (ROIs) of 2.5 cm diameter and composed of 123 voxels (Fig. 2). Mean ADC and standard deviation (SD) were computed.

According to the equations in Table 1, we computed the measurement repeatability (R), estimated by the coefficient of variation (CV_R_) and the repeatability coefficient (RC_R_), the accuracy (ADC Bias estimate), ADC noise estimate and b-value dependency.

**Table 1:** Definition of quality control metrics according to QIBA DW-MRI profile

	Metric	Definition
A	${\mathrm{CV}}_{\mathrm{R}}=100\%\ast \frac{\upsigma_{\mathrm{R}}}{\upmu_{\mathrm{R}}}$	CV_*R*_: Coefficient of variation (%) σ_R_: standard deviation (mm^2^/s) of each measurements means μ_R_ : mean of each measurements ADC means (mm^2^/s)
B	RC_R_ = 2.77 ∗ σ_R_	RC_R_ : repeatability coefficient (mm^2^/s)
C	ADC bias estimate = μ − DC_True_ ou $\%\mathrm{bias}=100\%\left(\frac{\upmu -{\mathrm{DC}}_{\mathrm{True}}}{{\mathrm{DC}}_{\mathrm{True}}}\right)$	DC_True_ : ADC=1,1.10^-3^mm^2^/s in 0°C water
D	$\mathrm{ADC}\ \mathrm{noise}\ \mathrm{Estimate}=100\%\ast \frac{\upsigma}{\upmu}$	σ : standard deviation of ADC values within the ROI (mm^2^/s) μ : mean ADC (mm^2^/s) within the ROI
E	$\mathrm{ADC}\ \mathrm{b}\ \mathrm{value}\ \mathrm{dependence}=100\%\left\Vert \frac{{\mathrm{ADC}}_{\mathrm{b}\ \min, \mathrm{b}2}-{\mathrm{ADC}}_{\mathrm{b}\ \min, \mathrm{b}1}}{{\mathrm{ADC}}_{\mathrm{b}\ \min, \mathrm{b}1}}\right\Vert$	bmin = b0 b1 = b600 b2 = b800
F	${\mathrm{SNR}}_{\mathrm{nDyn}}=\frac{\mathrm{Spatial}\ \mathrm{mean}\ \mathrm{pixel}\ \mathrm{value}\kern0.5em \mathrm{on}\ \mathrm{Signal}\ \mathrm{Image}}{\mathrm{Spatial}\ \mathrm{mean}\ \mathrm{pixel}\ \mathrm{value}\ \mathrm{on}\ \mathrm{Temporal}\ \mathrm{Noise}\ \mathrm{Image}}$	SNR_nDyn_ : Signal to Noise Ratio

The signal-to-noise ratio (SNR) was computed using formula F (shown in Table 1) and involved computing the ‘Temporal Noise Image’ from the diffusion mapping at b = 0, with a 2-cm circular ROI.

Results were compared to QIBA ‘s references values [16].

In addition, we analysed the planar spatial correlation of ADC measures in shifting ROIs along the x and y axis. The ADC reference value was measured at the image center using formula C (see Table 1). We used circular ROIs of 2.2-cm diameter and 2-cm shifts from the centre either to the right (x-axis) or to the bottom (y-axis) of the image.

### Measurement variability

#### SPHERE phantom study

A second phantom was used (NEMA NU2-2012 (PTW)), called SPHERE Phantom (Fig. 1, right side). The SPHERE phantom embedded six different spheres (diameters 10, 13, 17, 22, 28 and 37 mm), filled with room temperature water.

We simulated clinical conditions in using the cervical level of the routine whole-body MRI, i.e. axial DW SS EPI with b=50 and b=1,000 s/mm^2^, TR=10,384 ms, TE set to minimum (around 70 ms for all scans). Number of averages=2, parallel imaging factor=2, FOV=400*400 mm, contiguous 5-mm slice thickness, encoding frequency axis R/L. The phantom was laser centred, equidistant from all spheres. Four acquisitions were made at 1-day intervals. All values were averaged over 4 days.

**Fig. 3:**
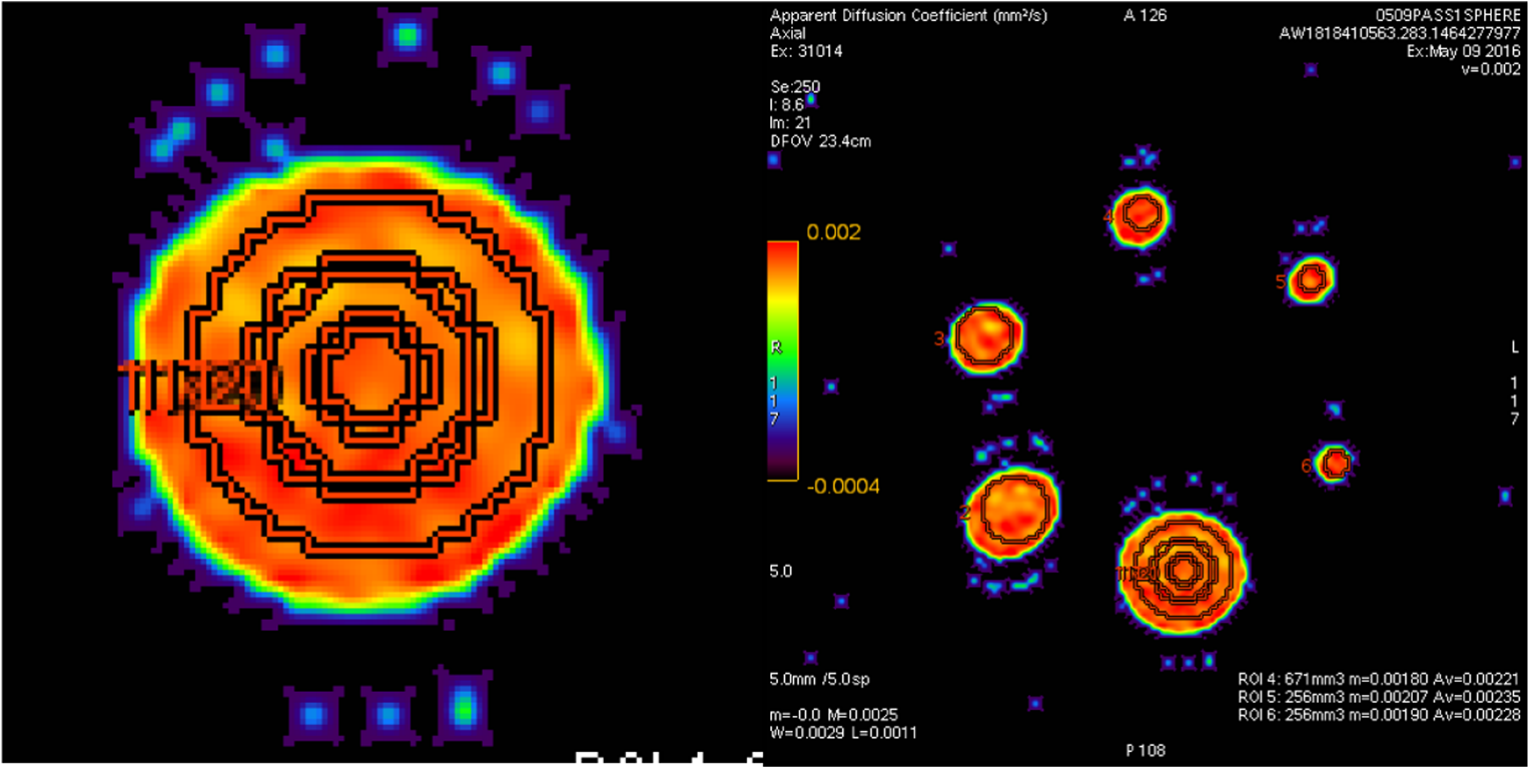
Measurements on the SPHERE phantom. **Left** Spherical VOIs of decreasing sizes centered on the largest sphere. **Right** Spherical volumes of interest (VOIs) centered on sphere of various sizes. VOIs diameters are set to 80 % of physical sphere’s diameters

ADC measures were obtained from spherical volumes of interest (VOIs) centred on spheres (Fig. 3).

The relative ADC error was computed for each sphere size, considering that the reference ADC value was from the 37-mm sphere. We analysed the correlation between VOI size and precision of measurements in computing the CV_R_. Additional analysis documented the measurement error, first in measuring bias, second in computing the CV_R_ through several concentric VOIs of decreasing size in the largest sphere, according to Table 1 (Formula A). Then partial volume effect was quantified by calculating the relative error within a VOI with a diameter equal to 80 % the diameter of a sphere compared to a VOI of identical size within the largest sphere. The mean and SD of ADC values were computed for all VOIs size.

### In vivo study

Informed consent was obtained from 13 healthy volunteers. Exclusion criteria were chronic disease, history or ongoing symptoms of infection like fever, cough, rhinorrhoea, dysphagia and odynophagia, history of cervical surgery, claustrophobia and all usual contraindications for MRI. Demographic status and smoking habits were recorded for the 13 volunteers. Volunteers were scanned using the same machine as the phantom study. The acquisition was performed with a neck phased-array coil and the volunteer was instructed to breath normally.

Technical settings of diffusion sequence for volunteers were identical to those of the SPHERE phantom.

Two readers assessed ADC values of lymph nodes: a senior radiologist with more than 6 years of experience in cancer imaging and a junior radiologist.

**Fig. 4:**
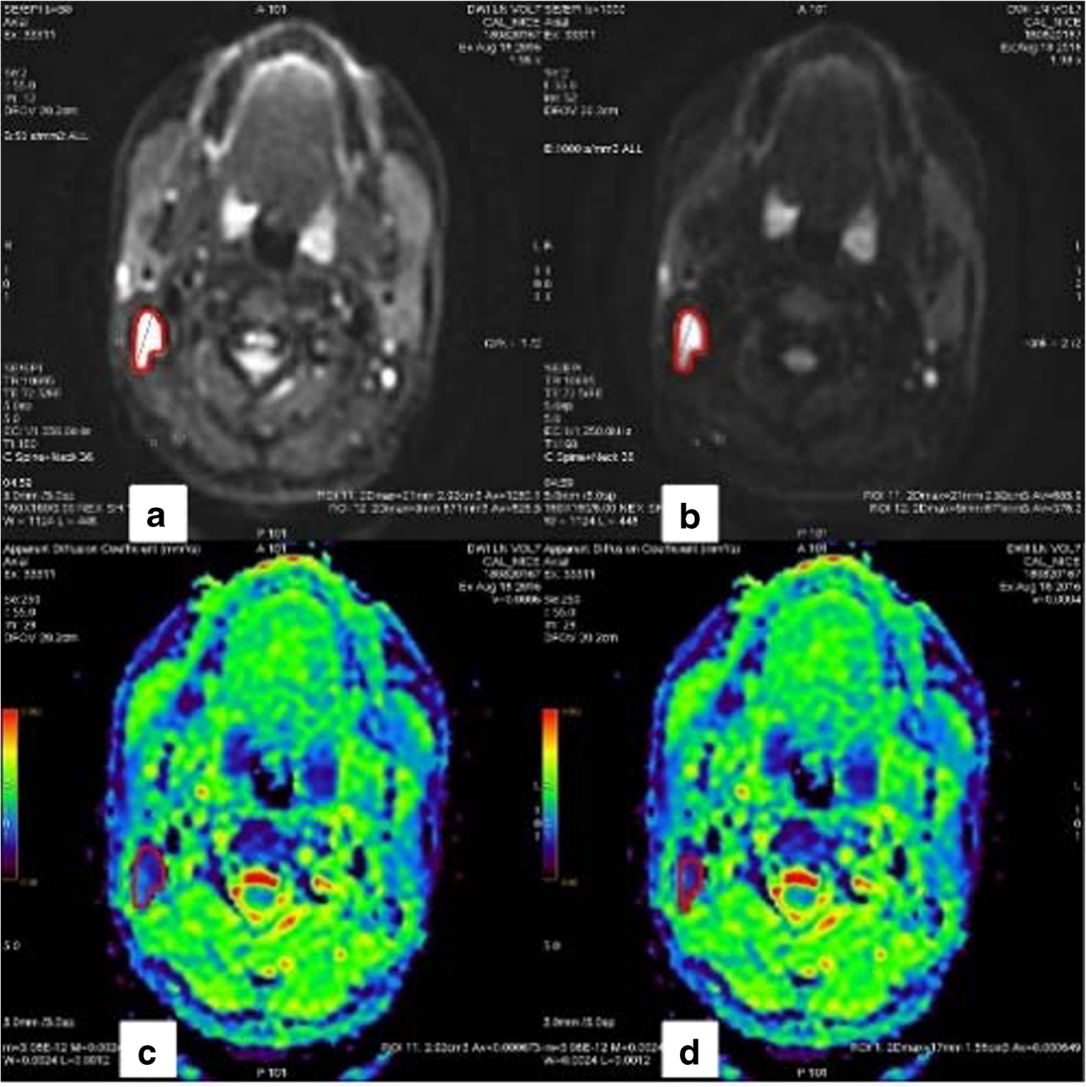
Measurements of cervical lymphnodes. Imaging of a healthy volunteer’s cervical lymph node. (**a**) Diffusion mapping at b = 50. (**b**) mapping at b = 1000 on which the volume of interest (VOI) is contoured before being exported to other series. (**c**) and (**d**) Mapping of the apparent diffusion coefficient. In red, the VOI is determined by operator 1 (**a**, **b** and **c**) and by operator 2 (**d**)

Lymph node volumes were manually segmented on the b1000 scan, and the graphic was exported to the ADC map (Fig. 4). At least four lymph nodes were selected per volunteer, including the largest. VOIs were segmented in delineating hyper-intense diffusion areas on b1000 scans while excluding lymph nodes hilum. Each node was segmented twice by each observer using the same acquisition with an interval of 7–60 days (mean 41 days) [21]. Mean and SD ADC values were recorded.

Inter- and intra-observer agreements were calculated according to the Bland Altman method using R CRAN software. Bias and limit of agreement (LoA) were computed. Inter- and intra-observer differences in segmenting lymph node volumes and ADC values were analysed using the sum of Wilcoxon rank for paired values test. A *p*-value < 0.05 was considered significant.

## Results

### Quality control test

Table 2 shows QIBA’s recommended limit values for repeatability, accuracy, precision and b-value dependency.

**Table 2:** Quality control after imaging the ICEWATER phantom

		QIBA claims	Results		
			b100	b600	b800
Repeatability	Coefficient of variation CV_R_ (%)	*< 1,5*	**2,5**	0,5	1,3
	Coefficient of repeatability RC_R_ (mm^2^/s)	*< 1,5.10* ^*-5*^	**7,96** ^**-5**^	**1,60.10** ^**-5**^	**4,16.10** ^**-5**^
Accuracy	*ADC Bias Estimate* (%)	*< 3,6*	3,4	**4,1**	**4,3**
Precision	*ADC Noise Estimate* (%)	*< 2*	**41,1**	**9,2**	**8,8**
**b-value dependency** (%) (b600-b800)		*< 2*	0,7		

Outcome of the quality control after imaging the ICEWATER phantom. The test was done with different b values as b0-b100, b0-b600 and b0-b800. Tests not meeting QIBA quality claims are displayed in bold

We found that SNR computed from diffusion scans at b = 0 was 17 1, lower than the recommended limit value (50 1).

**Fig. 5:**
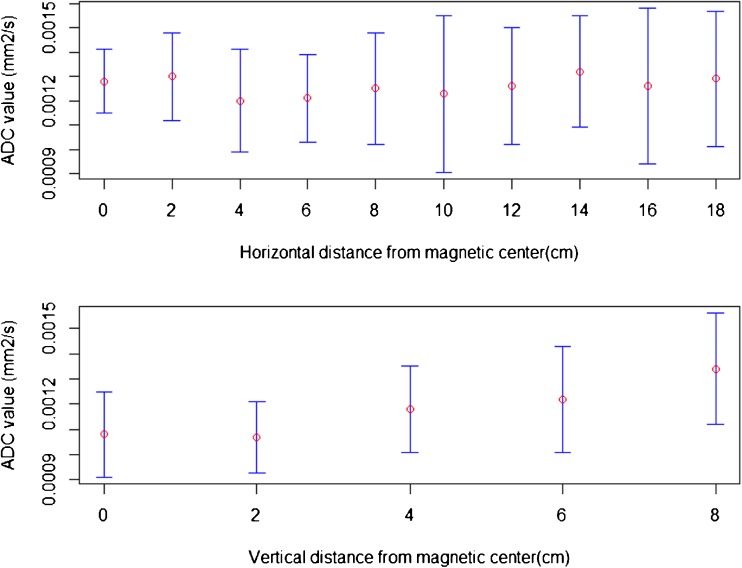
Spatial correlation of ADC. Top view apparent diffusion coefficient (ADC) changes according to the horizontal distance from magnetic centre. Horizontal axis distance in cm. Vertical axis ADC value in mm^2^/s. **Bottom view** ADC changes according to the vertical distance from magnetic center. **Horizontal axis** Distance in cm. **Vertical axis** ADC value in mm^2^/s

Variations in ADC measures relative to spatial positions are summarized in Fig. 5.

We found no significant correlation between ADC values and lateral shifts, but a significant correlation with vertical shifts (Pearson correlation coefficients, respectively ρ = 0.25, 95 % CI -0.45–0.76 and ρ = 0.95, 95 % CI 0.46–1).

We also found an increasing bias when shifting measurements from the scan isocenter (Table 3). The maximum 10 % error threshold recommended by QIBA was exceeded for ADC values measured at least 6 cm distant from the isocentre (Table 3).

**Table 3:** Spatial variations of apparent diffusion coefficient (ADC) measurements

Horizontal shift	**Distance from magnetic centre (cm)**	**0**	**2**	**4**	**6**	**8**	**10**	**12**	**14**	**16**	**18**	Pearson *ρ=0.25*
	*Shifted measurement* / Reference at magnetic centre (%)	0.0	1.6	-6.3	-5.5	-2.3	-3.9	-1.6	3.1	-1.6	0.8	
Vertical shift	**Distance from magnetic centre (cm)**	**0**	**2**	**4**	**6**	**8**						Pearson *ρ=0.95*
	*Shifted measurement* / Reference at magnetic centre (%)	0.0	-0.9	9.3	13.0	24.1						

Spatial variations of ADC measurements with respect to a reference VOI at center of the magnetic field. Top rows ADC measurements are shifted horizontally. Bottom rows ADC measurements are shifted to the bottom. In bold are the shift values corresponding to the distance from magnetic centre in cm.We found that ADC measurement did not change significantly when shifted right (Pearson coefficient=0.25). In opposite, ADC values increased when measurements were shifted vertically (Pearson coefficient=0.95)

### Reliability analysis

#### SPHERE phantom study

Firstly, our analysis showed that when VOIs are set within spheres of decreasing size, relative error and measurements variability of ADC measurements increased (Table 4). Secondly, we found no significant mean ADC difference for VOIs of decreasing sizes set within the largest sphere. In thoses case, we found less than 2 % error between the largest and smallest VOIs.

**Table 4:** Scaling effect of apparent diffusion coefficient (ADC) measurements

True sphere’s diameter (mm)	**37**	**28**	**22**	**17**	**13**	**10**
*Diameter of measured VOI’s (mm)*	*30*	*22*	*18*	*14*	*10*	*10*
Mean ADC over 4 days (.10^-3^ mm^2^/s)	2,05	2,07	2,13	2,15	2,31	1,97
Relative error of ADC measurements (%)	0 *(ref)*	0,98	3,9	4,76	12,8	-3,9
Coefficient of variation (%)	0,4	0,39	1,01	2,1	2,33	2,84

Repeated measurements of SPHERE Phantom images were performed over 4 days. Spheres of different sizes were measured. Top row (in bold) True size of spheres of interest. Second row Diameter of VOIs centered on spheres of interest. Third row For each sphere, measurements have been repeated four time over four days. Mean ADC values have been computed. Fourth row Relative error (%) with respect to the VOI set into the largest sphere. Bottom row Coefficient of variation (%) of repeated measurements over four days.

To be noted Regarding right side column, unlike for other measurements, true size sphere and VOI have same size (10mm) because of the sampling limit (8 Voxel into the VOI). As a consequence, a nonlinear effect has been observed Decrease of the Relative error and the mean ADC value. This point is further developed in Table 5

**Table 5:** Differential measurements: Largest sphere Vs spheres of smaller size

True sphere’s diameter (mm)	**37 vs 28**		**37 vs 22**		**37 vs 17**		**37 vs 13**		**37 vs 10**	
*Diameter of measured VOI (mm)*	*22 vs 22*		*18 vs 18*		*14 vs 14*		*10 vs 10*		*10 vs 10*	
Number of voxels sampling the measured VOI	*136*		*92*		*21*		*8*		*8*	
Mean ADC measurements (.10^-3^ mm^2^/s)	2,01	2,06	2,01	2,11	2,04	2,2	2,04	2,38	2,04	1,92
|*ADC* Relative error|(%)	2,5		5,0		7,8		16,7		5,9	

Repeated measurements of SPHERE Phantom images were performed over 4 days. Spheres of different sizes were measured. Top row (in bold) True size of spheres of interest (first value is the size of reference sphere, second value the size of sphere of interest). Second row Diameter of measured VOIs centered on spheres. Third row Number of voxel sampling VOIs. Fourth row For each sphere, measurements have been repeated four time over four days. Mean ADC values have been computed (first value is the size of reference sphere, second value the size of sphere of interest). Bottom row Relative error (%) with respect to the VOI set into the largest sphere as a reference.

Correlation and variability analysis of ADC measurements with VOI size seemed to indicate a significant partial volume effect. Partial volume effect was visually confirmed on images.

#### In vivo study

Thirteen volunteers were included in the in vivo study. Age ranged from 22 to 50 years (mean 32.4), and gender ratio (M/F) was 38.5 %. Two volunteers were active smokers or recent ex-smokers (15.4 %). Overall, 54 cervical lymph nodes were selected for analysis mainly on carotid-jugular sites, with a mean volume of 1 cm^3^ (Appendix 1).

The mean value of measured ADC was 0.87 × 10 -3 mm^2^/s (0.66–1.28 .10^-3^ mm^2^/s, SD was 0.12 .10^-3^ mm^2^/s). We found a significant difference between the average ADC values measured by readers 1 and 2 (0.84.10^-3^ and 0.90.10^-3^ mm^2^/s, respectively, *p* <0.0001).

The inter-reader analysis showed a relative bias of -5.5 %, LoA was [-18.8 %; 7.7 %]). The absolute bias was 0.045 10^-3^ mm^2^ / s, LoA was [-0.146; 0.056]).

We found a significant difference in average segmented volumes between readers 1 and 2 (respectively 1.18 +/- 0.94 cm^3^ and 1.92 +/- 1.23 cm^3^, *p* <0.0001). There was a low correlation between measurement differences in terms of average ADC and volume segmentation (R^2^ = 0.37) by the two observers.

Intra-observer analysis showed, respectively, for readers 1 and 2, a relative bias = 0.6 %; LoA=[-9.2 %; 10.4 %] and relative bias = 0.5% ; LoA=[-8.8 %; 7.7 %].

Using the Beaumont et al. method [22] and based on our intra-observer reproducibility parameters, we can estimate that on longitudinal studies under strict reproducible conditions (same patient, same reader), a meaningful relative change of ADC value should be outside the range [-13 %; + 15% ].

## Discussion

Our QC results showed good compliance with QIBA metrics, except for ADC bias estimate, which was slightly above the limit, and with a variability of about 9 %. Results were independent of the value of b.

We questioned if the main part of error was due to our phantom design featuring a large off-axis volume of water and thermally suboptimal materials. Using repeated imaging of the phantom, we found, however, a good repeatability, suggesting acceptable thermal equilibrium.

SNR was also lower than QIBA’s recommendation, but Malyarenko et al. [18] reported that low SNR has no impact on ADC assessments. Very low SNR without adequate post-processing would probably alter measurements as most software (including the one we used) compute ADC images in thresholding/removing low intensity voxels.

We highlighted a correlation between ADC measurement error and the distance of ROIs from the magnetic centre. The error increased with bottom-shift (up to 24 % when located 8 cm out of isocentre). Conversely, with regard to lateral-shift we found no correlation with the magnitude of errors. This result can be explained by non-uniformity of gradient-fields.

As we found a correlation between variability of ADC assessments and contour segmentations, we concluded that partial volume effect was a major contributor to the variability.

**Fig. 6:**
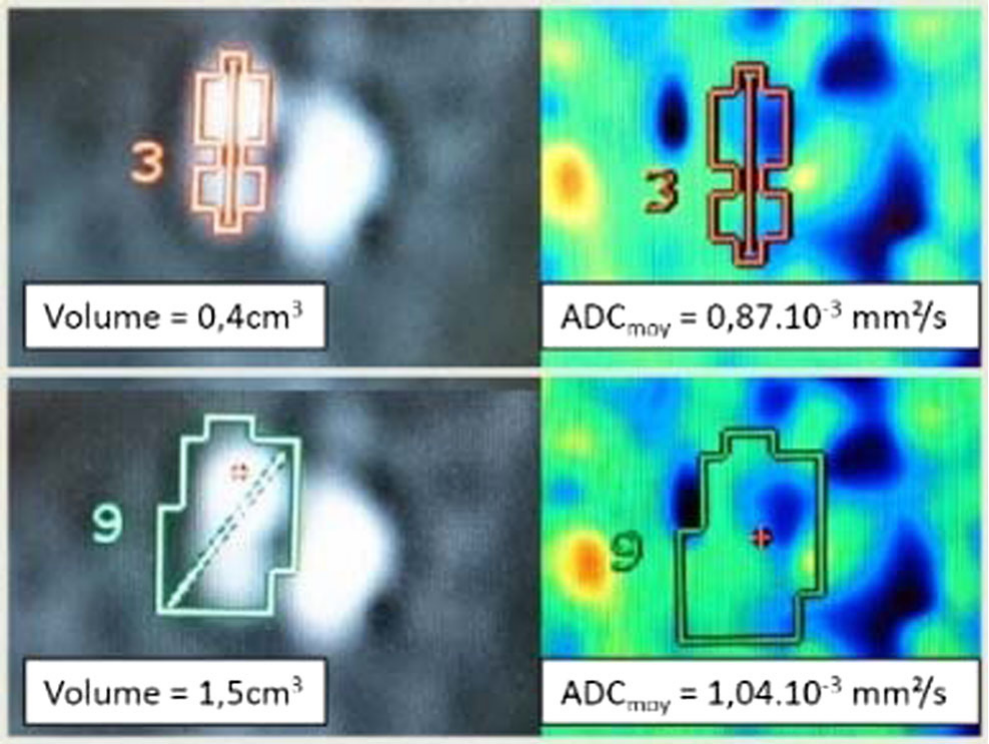
Example of inter-observer discordance in terms of volume and apparent diffusion coefficient (ADC). Example of inter-observer discordance in terms of volume and ADC on a level II lymph node. **Top row** First reader’s measurements. **Bottom row** Second reader’s measurements. **Left** b1000 diffusion maps where volumes of interest (VOIs) are drawn. **Right** corresponding ADC maps. Note: The heterogeneity of the node’s environment featuring areas of high ADC values (green and red in the right images) without clear correspondence on the diffusion image

A visual review of outliers in clinical data confirmed high variation of signal intensity in the tissue surrounding these lymph nodes. Consequently, even a small variation in segmentations led to a significant modification of ADC assessments (Fig. 6)

We recommend measuring ADC by drawing ROIs smaller than the anatomical limits of the area of interest. How to optimize segmentation margins must be further investigated.

We found excellent repeatability and good reproducibility. This suggest that, if ADC intended to evaluate response to treatment, changes inferior to [-13 %; +15 %] may not be clinically relevant. However, longitudinal reproducibility would require further clinical studies to take into account all variability factors.

According to our dataset, the averaged ADC value for healthy subject's cervical lymph nodes was 0.87.10^-3^ ± 0.12 .10^-3^ mm^2^/s.

Our results are well supported by the literature.

Regarding the correlation between variability of ADC assessments and contour segmentation, heterogeneous segmentation methods are available but several studies documented the reproducibility issues [23–25] affecting these methods. These different approaches are also reported as cumbersome and time-consuming [26].

Specific phantom studies have shown that gradient-field error would be scanner-dependent [27] and not significant within 4 cm from the isocentre, explaining the good reproducibility of our ADC measurements and in other multicentric studies. On multiple scanners, measurements at 12 cm from the isocentre showed an average error of -20 % according to vertical shift and +7 % horizontally.

Unlike our observation of an ADC value of 0.87.10^-3^ ± 0.12 .10^-3^ mm^2^/s in healthy subject's cervical lymph nodes, Kwee et al. [12] reported a range of [1.15 10^-3^ mm^2^/s; 1.18 10^-3^ mm^2^/s], with similar intra- and inter-observer variabilities. A review of 12 studies including more than 1,200 benign lymph nodes report ADC values ​of 0.302 ± 0.062.10^-3^ mm^2^/s in inflammatory cervical nodes [28] and 2.38 ± 0.29.10^-3^ mm^2^/s for abdominal nodes [29]. Kwee et al. concluded that disparity of results could be due to the various segmentation methods used.

Our results for non-diseased ADC values overlap with metastatic or lymphomatous lymph nodes ADC measures [0.410 ± 0.105 .10^-3^ mm^2^/s; 1.84 ± 0.37 .10^-3^ mm^2^/s] as reported by other groups [28, 29]; however, the ADC values we found match with other non-diseased ADC studies. A radiological-pathological correlation study by Vandecaveye et al. [30] on 331 cervical lymph nodes proposed an ADC threshold of 0.94 10^-3^ mm^2^/s for detecting node malignancy featuring a specificity of 94 %. According to this threshold, 72 % of our data would have been misclassified. The use of ADC values to assess cervical lymph nodes malignancy does not reach a consensus.

Other variability factors are described in the literature.

1. Inter-scanner variability. Some authors [18] report less than 3 % variability while others [31] conclude that 80 % of scanners featured less than 5 % error. Another group [32] reports that the CV_R_ was 1.5 % on phantoms, and less than 4 % for cerebral parenchyma. Impact of acquisition parameters has also been investigated [33–35].
2. Impacts of post-processing software were documented by Zeilinger et al. [36], reporting up to 8 % variation when ADC was processed by four different types of software. This limitation precludes longitudinal assessment of patients across different centers.

In order to address the limitations we found, variability can be minimized by standardizing ADC assessments. Efforts from scanner manufacturers are needed [8] to ease the calibration of diffusion sequences. Also, initiatives from the scientific community and technological improvements are expected to avoid sequences artifacts and systematic errors. Correction of non-linear gradient fields, one of the relevant issues, is the focus of ongoing research [37, 38].

Design of standardized validation methodologies and systematic quality control [39] are key to reach acceptable levels of compliance. To this end, a commercial version of diffusion phantom has been developed by QIBA [40] and an automatic quality control software is under development.

From early quantitative evaluation of diffusion, other approaches have emerged.

IVIM (Intra-Voxel Incoherent Motion) is aiming to split the different components of ADC [41]. Some authors indicate that pure molecular diffusion would be more meaningful than ADC as being independent from the perfusive component [42]. Therefore, IVIM would enable assessing tissue for which ADC is conceptually limited. Other studies develop alternative diffusion-derived QIBs in using the non-Gaussian distribution of diffusion kurtosis, Q-ball imaging and spectrum analysis in particular.

These approaches also require metrological analysis before scientific validation can be obtained, although the scientific literature shows potential added value of ADC.

The physiopathology of ADC changes still remains unknown. Some malignant tumours may feature increased ADC with respect to healthy tissue, either by spontaneous necrosis or cystic transformation, or by the destruction of a parenchyma with spontaneously low ADC. Some benign tumours may lead to ADC restriction; this is the case with Warthin tumours of salivary glands due to their cellular wealth, which is superior to the normal salivary tissue [43].

Lastly, microscopic changes triggered by anti-cancer treatments may interfere with ADC assessment follow-up [44] post-chemotherapy cytotoxic oedema leading to increased restriction of ADC despite a therapeutic response, and delayed fibrosis that may lead to suspicion of recurrence by dropping the ADC. Other confounding factors such as the appearance of extracellular oedema by hyper-hydration or by regional venous obstacles may also mislead the evaluation of therapeutic responses.

We found several limitations in our study. Phantom designs are not optimal and may have biased our results, limiting interpretations. First, regarding our ICEWATER phantom, even if the thermal equilibrium seemed satisfactory, a 1 °C error could lead to a 2.4 % ADC error, explaining the slightly higher value found compared to QIBA recommendation. Second, with regard to the SPHERE phantom: (1) without thermal control, measurements were relative, limiting generalizability, (2) comparison of distant ROIs without gradient field correction was prone to bias, and (3) analysis of partial volume effect can be affected by phantom material, gas and features. Third, we did not evaluate the impact of artifacts [45]. QIBA recommends the use of corrective methods for liver and kidney analysis [8], although other groups [46, 47] report no improvement in using such methods. Fourth, the monocentric design of our study limits the generalizability of our results.

At this time, variabilities from different sources preclude a larger adoption of the ADC biomarker even though it is an important advance in cancer imaging. Generalizing quality controls and standardization of measurements is crucial to overcome these ADC variability issues.

## Electronic supplementary material

ESM 1 (DOCX 20 kb)

## Abbreviations

ADC: Apparent diffusion coefficient
CV_R_: Coefficient of variation
FOV: Field of view
GE: General electric
IVIM: Intra-Voxel Incoherent Motion
LoA: Limit of agreements
MRI: Magnetic resonance imaging
PET: Positron emission tomography
QC: Quality control
QIB: Quantitative Imaging Biomarker
QIBA: Quantitative Imaging Biomarker Alliance
RC_R_: Repeatability coefficient
ROI: Region of Interest
RSNA: Radiological Society of North America
SD: Standard deviation
SNR: Signal-to-noise ratio
T: Tesla
