# Supplementary material for: How reliable are ADC measurements? A phantom and clinical study of cervical lymph nodes
Source: Eur Radiol. 2018 Feb 23;28(8):3362–71. doi: 10.1007/s00330-017-5265-2 (PMC6028847; doi:10.1007/s00330-017-5265-2)
Supplement: Supplementary file 1 — (DOCX 20 kb) [file 330_2017_5265_MOESM1_ESM.docx]

**Supplementary material**

**Appendix 1: Description of included lymphnodes.**

| **Nodes localization** | **Level I** | **Level II** | **Level III** | | **Level IV** | **Level V** |
| --- | --- | --- | --- | --- | --- | --- |
| **Number (%)** | 1 (1.9%) | 45 (83.3%) | 3 (5.6%) | | 0 | 5 (9.3%) |
| **Mean volume (cm^3^)** | 0.88 | 1.72 | 0.83 | | 0 | 0.66 |
| **Mean ADC (.10-^3^ mm²/s) ± SD** | 0.76 | 0.87 +/-0.13 | 0.93 +/-0.18 | | 0 | 0.86 +/-0.03 |
| **Active or recently weaned smoking** | **Yes** | | | **No** | | |
| **Number (%)** | 2 (15.4%) | | | 11 (84.6%) | | |
| **Mean ADC (.10^-3^ mm²/s) ± SD** | 0.84 +/- 0.12 | | | 0.88 +/- 0.13 | | |
| **Gender** | **Male** | | | **Female** | | |
| **Number (%)** | 5 (38.5%) | | | 8 (61.5%) | | |
| **Mean ADC (.10^-3^ mm²/s) ± SD** | 0.81 +/- 0.09 | | | 0.91 +/- 0.13 | | |
| **Age** | **< 35 years** | | | **> 35 years** | | |
| **Number (%)** | 10 (76.9%) | | | 3 (23.1%) | | |
| **Mean ADC (.10^-3^ mm²/s) ± SD** | 0.86 +/- 0.10 | | | 0.92 +/- 0.18 | | |
